# Thematic development of research on patient safety: An analysis with the science mapping technique

**DOI:** 10.14744/nci.2021.87847

**Published:** 2022-04-13

**Authors:** Umut Beylik, Tuncay Palteki

**Affiliations:** 1 Department of Health Management, University of Health Sciences, Gulhane Faculty of Health Sciences, Ankara, Turkey; 2 Department of Emergency Aid and Disaster Management, Biruni University Faculty of Health Sciences, Istanbul, Turkey

**Keywords:** Bibliometric analysis, patient safety, science mapping, SciMAT, thematic development

## Abstract

A large amount of scientific literature is forcing those who want to study the subject deeply. In general, the number of articles available exceeds thousands. It has become challenging both to dominate the current concept and to see the relationships between the developments that feed the concept. Visualization techniques based on bibliometric data help to gain an overview of the literature on complex research topics. The aim of this study is to examine the topic of “patient safety” with a bibliometric analysis program. The data of 8372 articles obtained from the Web of Science database were analyzed with SciMAT2 software. Before the analysis, general findings regarding the raw data were brought to the fore. The strategic diagram and thematic development map have been analyzed in terms of 10-year periods covering the past 30 years. Five motor themes (“contrast agent,” “adverse events,” “program,” “safety,” “prostatectomy”) were identified in 1990-1999, four motor themes (“infection control,” “hospital-acquired infections,” “adverse drug events,” “culture”) in 2000-2009 and nine motor themes (“patient safety,” “education,” “climate,” “system,” “mortality,” “operating room,” “validity,” “burnout,” “primary care”) in 2010-2019. The number of motor themes increased during the period and adverse events, which were the active subjects of the early periods, were replaced by new concepts (such as climate, primary care, and burnout) over time. Bibliometric visualization tools make it possible to analyze the literature consisting of a large number of articles. This approach facilitates a person’’s understanding of a complex research topic such as patient safety and ensures that they are aware of new research directions or alternative research priorities.

**P**atient safety is a multifactorial, multidimensional, and interdisciplinary research subject [1]. After the report “To Err Is Human: Building a Safer Health System” of the Institute of Medicine in 1999 [2] and the study “Crossing the quality chasm: a new health system for the 21^st^ century” published in 2001 [3], the concept of “patient safety” has gained a great deal of attention. One of the institutions that feed and classify the concept of patient safety is WHO. As a framework, WHO developed an international classification system for patient safety in 2009 (WHO, 2009) [4].

Due to its complex character and its relation with other concepts, the number of publications on patient safety has increased over the years. The growing number of publications makes it difficult to look at patient safety literature with a comprehensive overview. When concepts such as equipment safety, safety culture, adverse events, error reporting, and medical errors are searched together in databases along with the notion of patient safety, the search results result in hundreds of thousands of articles.

When bibliometric studies on patient safety were examined; it is seen that concepts such as medication errors and adverse errors [5], the relationship of health information technologies with patient safety and quality [6], patient safety and risk management [7], adverse events in intensive care units [8], patient safety culture [9] and safety leadership [10] were studied. The only study that examined the concept of patient safety from a holistic perspective and in all aspects is Rodrigues et al. [1], which was conducted in 2014. In this study, citation analysis and text mining operations were performed with bibliometric data using VOSviewer software. This present study examined patient safety data from a holistic perspective with SciMAT software and examined the main themes of patient safety and the transformation it has gone through over the years with strategic diagram analysis. This study has brought a different perspective to the subject with different analysis techniques using a different software. Besides, since the data of this study covers the period after 2014, it has a more comprehensive view. In the study, it was aimed to evaluate the development of publications on “patient safety” over time and scientific mapping of the subject was carried out.

## Methods

The data of the publications analyzed in the study were downloaded from the Web of Science (WOS) Core Collection database. On 25.09.2020, 8372 publications were reached during the search with the term “patient safety” on the “Title” tab in the WOS database and the data of these publications were downloaded in plain text format. The downloaded data were uploaded to the SciMAT program for scientific mapping analysis. As a result of the examination, 30 publications that do not contain year information and 44 publications of 1989 and earlier, which do not contain the data required for analysis, were excluded from the analysis. To see the development periodically in the analysis, 8298 publications are divided into the periods 1990–1999, 2000–2009, and 2010–2020. The analysis included 84 publications in the first period, 2009 in the second period, and 6205 in the third period.

In the analyzes, words were used as research units. Before the analysis, 9962 keywords used in publications were grouped by considering singular-plural, abbreviation, misspelling, and same meanings. As a result of the analysis, data reduction was made to ensure that the findings can be interpreted. In the analysis, “co-occurrence” in matrix type, “equivalence index” in normalization measurement, “simple centers algorithm” in cluster algorithm, “core mapper” options were used in the mapping. The h-index and the total number of citations were selected in quality measurement. “Inclusion index” options were used in the thematic development map and overlap map.

**Highlight key points**

• Approximately 45% of the studies on patient safety have been carried out in the USA.
• The most cited three patient-safety-related author/article topics are; communication and information transfer among physicians, information technology, and infection control.
• Patient-safety related motor themes have manifested themselves in the last decade as “patient safety,” “education,” “climate,” “system,” “mortality,” “operating room,” “validity,” “burnout,” and “primary care.”

Research findings were evaluated visually with strategic diagrams, thematic networks, overlap maps, and thematic development maps. In the strategic diagram, the emerging themes can be located in four different areas according to their centrality and intensity levels: Centrality is related to the outer relations of the theme, and themes shift to the right side in the diagram as the level of relationship with other themes increases. Density is related to the inner relations of the theme, and themes with increasing levels of relationship in themselves move upward in the diagram. The characteristics of the groups with these themes are as follows:

• Themes with high centrality and density in the upper right area where motor themes are located
• Themes with high centrality but low density in the lower right area where basic and transformational themes are located
• Themes with low centrality but high density in the upper left area where developed and isolated themes are located
• In the lower left area where emerging or disappearing themes are located, there are themes with low centrality and density.

In thematic networks where the relationships between themes in the theme cluster are evaluated, the size of the themes varies according to the number of publications. The thickness of the lines varies according to the degree of the relationship. In the overlap map where the numerical development of the keywords used in the publications is evaluated periodically, the number and percentage of keywords used in the previous period and transferred to the next period, the number of keywords that have just started to be used, and the number of keywords that were used in the previous period but not in the relevant period can be seen. In the thematic development map, the relationships of the themes between periods are evaluated. Straight lines on the map indicate that the same keywords are shared with the theme names. Dashed lines, on the other hand, indicate that common words are shared except for the theme names. The thickness of the lines varies according to the degree of the relationships, and the size of the themes depends on the number of publications [11–15].

## Results

We evaluated the findings under two headings. Firstly, the number of publications by years, the number of publications by country, the most cited, most productive authors, and the most used keywords in the research are given under the heading of general findings. Secondly, basic themes and strategic diagrams were analyzed according to the results of SciMAT software.

**Figure 1.**
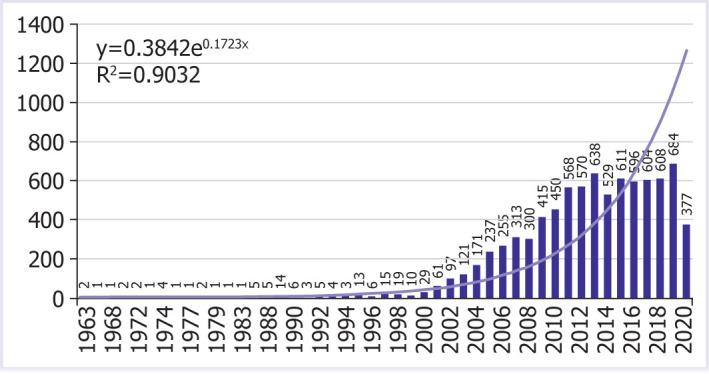
Number of publications by years.

General Findings; The distribution of the publications downloaded from the WOS database for analysis by years is presented in Figure 1. According to these findings, it is seen that there were very few publications on the subject before 2000, that the number of publications between 2000–2013 increased in general, and there was a recession between 2014–2018.

**Figure 2.**
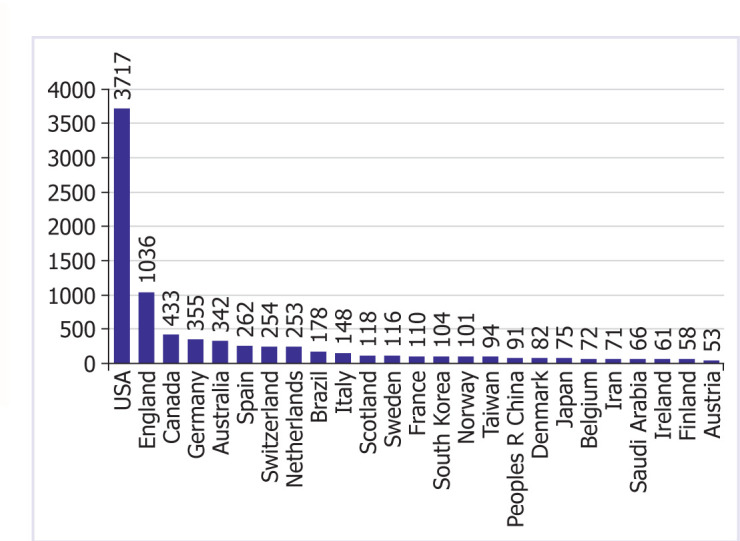
Number of publications by country (top 25 countries).

The distribution of publications downloaded from the WOS database by country is presented in Figure 2. According to these findings, the most publications were made by the USA, and this country was followed by the UK, Canada, Germany, and Australia. Turkey ranks 28^th^ with 51 publications. English, German, and Spanish were the most widely published languages of publications, and Turkish was ranked 10^th^ with 5 publications.

It was determined that the total number of citations of 8372 publications downloaded from the WoS database was 87,230. This number was 70,889 when self-citations were excluded and h index value of these publications was determined to be 111. The most cited publication information is included in Table 1. According to these findings, it is seen that the most cited publication (n=1070) is the publication named “Deficits In Communication And Information Transfer Between Hospital-Based and Primary Care Physicians - Implications for Patient Safety and Continuity of Care” published by Kripalani et al [16].

**Table 1.** Top 10 cited publications

Rank	Reference	Title	Total citations
1	Kripalani et al., 2007	Deficits in communication and information transfer between hospital-based and primary care physicians - Implications for patient safety and continuity of care	1070
2	Bates and Gawande, 2003	Patient safety: Improving safety with information technology	813
3	Burke, 2003	Infection control - A problem for patient safety	627
4	Carayon et al., 2006	Work system design for patient safety: the SEIPS model	557
5	Manser, 2009	Teamwork and patient safety in dynamic domains of healthcare: a review of the literature	510
6	Blendon et al., 2002	Patient safety: Views of practicing physicians and the public on medical errors	481
7	Trzeciak and Rivers, 2003	Emergency department overcrowding in the United States: an emerging threat to patient safety and public health	452
8	Rogers et al., 2004	The working hours of hospital staff nurses and patient safety	439
9	Gaba and Howard, 2002	Patient safety: Fatigue among clinicians and the safety of patients	421
10	Khuri et al., 2008	Successful implementation of the department of Veterans Affairs’ National Surgical Quality Improvement Program in the private sector: The patient safety in surgery study	414

Information on the most productive authors is presented in Table 2. According to these findings, it is seen that most publications were made anonymously. Pronovost PJ and Schwappach D were among the most published authors after anonymous. The most used words among the keywords in the publications analyzed in the SciMAT program are presented in Table 3. According to these findings, the most frequently used words were “patient safety” and “care” and “adverse events.”

**Table 2.** The 10 most productive authors

Rank	Author name	Document number
1	Anonymous	160
2	Pronovost PJ	66
3	Schwappach D	58
4	Vincent C	45
5	Rosen AK	41
6	Bates DW	40
7	Wachter RM	37
8	Wagner C	36
9	Sevdalis N	31
10	Romano PS	29

**Table 3.** The most used keywords in the research

Rank	Word group	Number of uses
1	Patient safety	2117
2	Care	754
3	Adverse events	730
4	Quality	640
5	Errors	610
6	Medical errors	591
7	Health care	552
8	Hospitals	345
9	System	298
10	Impact	293
11	Outcomes	283
12	Management	279
13	Culture	277
14	Quality improvement	273
15	Nurses	272
16	Education	254
17	Mortality	252
18	Performance	246
19	Communication	224
20	Climate	223

**Figure 3.**
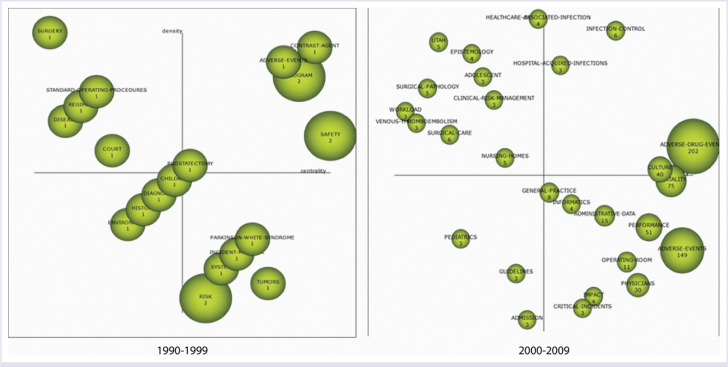
Strategic diagram (1990–1999; 2000–2009).

The strategic diagram for the period 1990–1999, which emerged as a result of the analysis, is given in Figure 3. During this period, 19 themes emerged. Five of these themes are motor themes (“contrast agent,” “adverse events,” “program,” “safety,” “prostatectomy”), 5 of them are isolated and advanced themes (“surgery,” “standard operating procedures,” “regimen,” “disease,” “court”), 5 of them are basic and transformational themes (“Parkinson white syndrome,” “incident monitor,” “system,” “tumors,” “risk”), 4 of them are emerging or disappearing themes (“children,” “diagnosis,” “history,” “environment”).

The strategic diagram for the period 2000–2009 obtained as a result of the analysis is shown in Figure 3. During this period, 27 themes emerged. Four of these themes are motor themes (“infection control,” “hospital acquired infections,” “adverse drug events,” “culture”), 10 of them are isolated and advanced themes (“healthcare associated infection,” “utah,” “epistemology,” “adolescent,” “surgical pathology,” “clinical risk management,” “workload,” “venous thromboembolism,” “surgical care,” “nursing homes”), 10 of them are basic and transformational themes (“mortality,” “general practice,” “informatics,” “administrative data,” “performance,” “adverse events,” “operating room,” “physicians,” “impact,” “critical incidents”), 3 of them are emerging or disappearing themes (“pediatrics,” “guidelines,” “admission”).

According to the findings related to the 2000–2009 period themes, 202 publications were made related to the theme of “adverse drug events,” one of the motor themes of this period. It was determined that the total number of citations of these publications was 9.521 and the h-index value was 51. After the “Adverse drug events” theme, it is seen that the theme with the most publications, the most citations and the highest h index is the “adverse events” theme.

**Figure 4.**
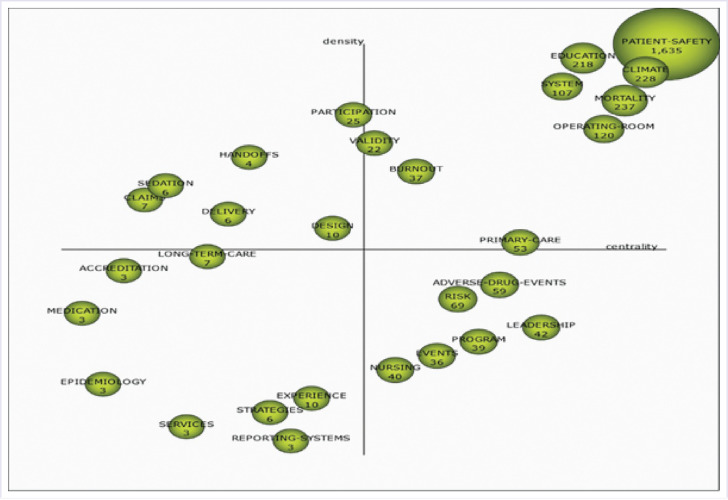
Strategic diagram (2010–2020 period).

The strategic diagram for the period 2010–2020 obtained as a result of the analysis is shown in Figure 4. During this period, 29 themes emerged. Nine of these themes are motor themes (“patient safety,” “education,” “climate,” “system,” “mortality,” “operating room,” “validity,” “burnout,” “primary care”), 6 of them are isolated and advanced themes (“participation,” “handoffs,” “sedation,” “claims,” “delivery,” “design”), 6 of them are basic and transformational themes (“adverse drug events,” “risk,” “leadership,” “program,” “events,” “nursing”), 8 of them are emerging or disappearing themes (“long term care,” “accreditation,” “medication,” “epidemiology,” “experience,” “strategies,” “services,” “reporting systems”).

**Figure 5.**
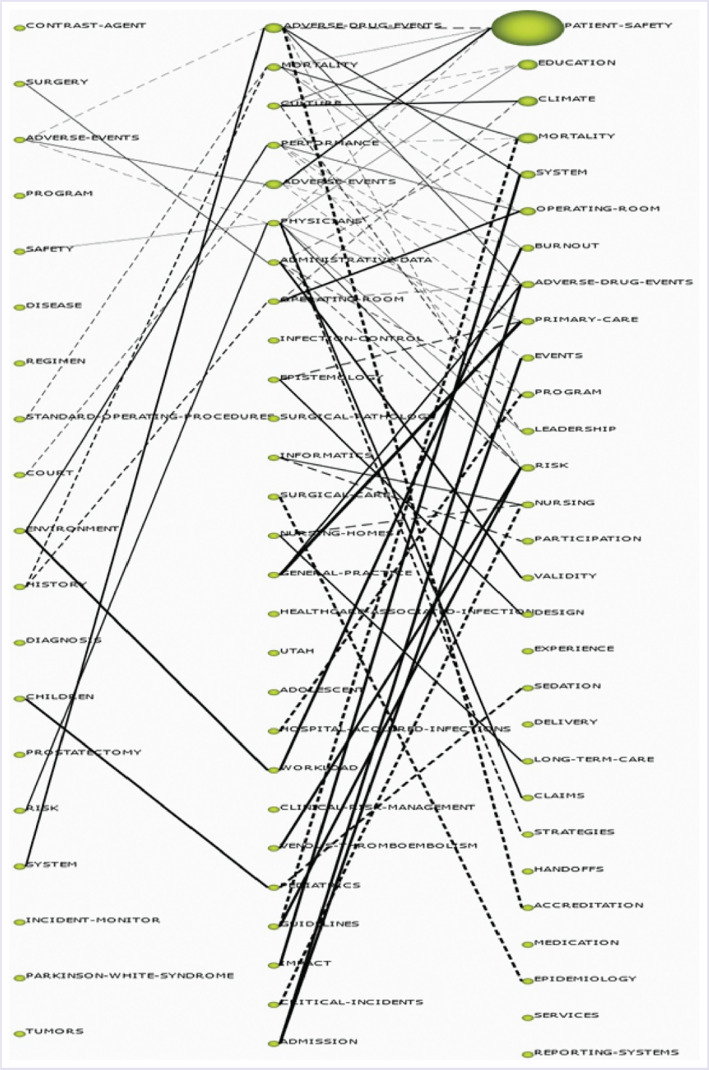
Overlap map.

The numerical development of keywords in publications evaluated in the study by periods is presented in Figure 5 in the overlap map. According to these findings, 46 (38%) of the 120 keywords used in the first term were also used in the second term, and a total of 2516 words were used in the second term together with the newly added words. 1.335 (53%) of the keywords in the second period were transferred to the third term and a total of 8017 words were used in the third period with the newly added words.

**Figure 6.**
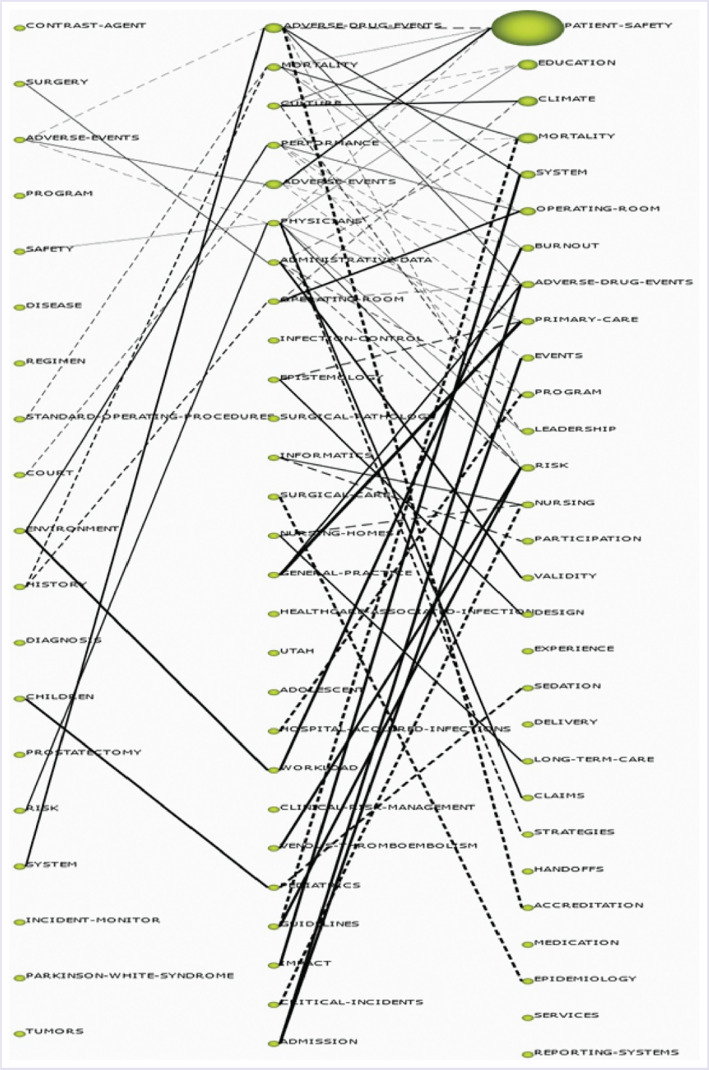
Thematic development map.

Thematic development map, in which the relationships between themes in the analyzed periods are evaluated, is presented in Figure 6. The highlights of these relationships are presented below. The “adverse drug events” theme, which was among the motor themes in the first period, took place among the basic and transformational themes in the second period. The “adverse drug events” theme, which is associated with the themes of “adverse events” and “system” from the first period, is related to the themes of “patient safety,” “system,” “risk” and “accreditation” from the last period. The “adverse events” theme, which is among the motor themes in the first period and among the basic and transformational themes in the second period, showed a strong relationship with the “patient safety” theme from the last period.

The theme of “physicians” in the second period showed a relationship with the “adverse events” and “safety” themes from the first period and the “patient safety,” “education,” “primary care,” “leadership,” “claims” themes from the last period, as well as the “risk” theme in the first and last period. There is a strong relationship between the “administrative data” theme, which is associated with the “surgery” theme from the first period, and the “validity” theme from the last period. The “operating room” theme, which is among the basic and transformational themes in the second period and one of the motor themes in the last period, shows a strong relationship between itself. However, it shows relations with the themes of “history” from the first period, “climate” and “adverse drug events” from the last period.

It is seen that the “surgical care” theme in the second period frequently shared other keywords other than the basic keywords with the “epidemiology” theme from the last period. It is seen that the “nursing home” theme in the second period has a stronger relationship with the “long term care” theme in the recent period and it is also associated with the theme of “nursing.” At the same time, it is seen that the theme of “nursing” is related to the themes of “informatics” and “critical incidents” from the second term.

The theme of “general practice” that emerged in the second period shares the basic keywords with the theme of “primary care” and “adverse drug events” from the last period. The “workload” theme, which is associated with the “environment” theme from the first period, also shows a strong relationship with the “burnout” theme from the last period. It is observed that the “risk” theme, which took place in the last period, had a strong relationship with the “venoes thromboembolism” and “guidelines” themes from the second period, and at the same time, the “guidelines” theme was associated with the “mortality” theme from the last period.

It is seen that the “pediatrics” theme, which is associated with the “children” theme from the first period, is associated with the “sedation” theme from the last period. It is observed that the “admission” theme in the second period showed a strong relationship in the form of basic keyword sharing with the themes of “events” and “adverse drug events” from the last period.

## Conclusion and Recommendations

The results of the bibliometric analysis made through the SciMAT program in the context of patient safety show that there is a stagnation in studies conducted in recent years (2014–2018). According to the findings, most of the studies were conducted in the USA and published in English, and “patient safety,” “care,” “adverse events,” “quality” and “errors” were the most used phrases in the studies.

According to the findings in this study, the engine themes focused on “adverse event” and “safety” in the 1990s. In the 2000s, it evolved into “infection control,” “hospital-acquired infections,” “adverse drug events,” “culture.” In the 2010s, it emerged as “patient safety,” “education,” “climate,” “system,” “mortality,” “operating room,” “validity,” “burnout,” “primary care.” It is seen that the issues that were not included in the studies in the 1990s started to be taken into consideration in the following years and issues such as patient safety, education, system, climate were included in the studies. According to the thematic development map data, it reveals that the adverse event theme has a strong relationship with the “patient safety” theme.

It is seen that studies conducted in the context of patient safety have improved over time, and themes and words that were not previously included in the studies have begun to be taken into account over the years. It is noteworthy that one of the expressions in which the theme of patient safety is most often used together and the strong connection between them is “advertise event.” However, the theme of “quality improvement” is another theme that has a strong connection with patient safety. Studies conducted in the context of patient safety draw attention to the necessity of efforts to prevent adverse events in health institutions and to improve the quality of health care, and to increase the education and knowledge levels of patients and healthcare professionals. Besides, it was underlined that the risk management mechanisms should be operated by health administrations and the prevention activities should be carried out before the incident occurs in the institutions. The importance of analyzing the incident reports obtained with the security reporting systems to be established, and making the necessary arrangements were emphasized in this study. It should not be forgotten that every precaution to be taken to ensure patient safety can be a means of saving a life.

As a result, a large amount of literature can be analyzed using bibliometric data. Visualization of these data using tools such as SciMAT makes it possible to obtain a broad overview of the structure of the literature on a particular topic. This approach can be very useful for better understanding of a complex research topic such as patient safety. Other complex multidimensional research areas (such as evaluating health technologies) can be analyzed similarly. This method of literature analysis can help suggest new research directions or alternative research priorities.
